# Prenatal exposure to perfluoroalkyl substances and inflammatory biomarker concentrations

**DOI:** 10.1097/EE9.0000000000000262

**Published:** 2023-07-25

**Authors:** Jana Palaniyandi, Jennifer E. Bruin, Premkumari Kumarathasan, Susan MacPherson, Michael M. Borghese, Jillian Ashley-Martin

**Affiliations:** a Department of Biology and Institute of Biochemistry, Carleton University, Ottawa, Ontario; b Environmental Health Science and Research Bureau, Health Canada, Ottawa, Ontario

**Keywords:** Cohort, Inflammatory biomarkers, Perfluoroalkyl substances, Pregnancy

## Abstract

Per- and polyfluoroalkyl substances (PFAS) are persistent environmental contaminants that induce immunotoxicity in experimental studies; however, epidemiological evidence—particularly during pregnancy—is scarce. We quantified associations between first trimester plasma perfluorooctanoic acid (PFOA), perfluorooctane sulfonate (PFOS), and perfluorohexane sulfonate (PFHxS) concentrations and third trimester concentrations of inflammatory biomarkers and determined if these associations were modified by fetal sex.

**Methods:**

We analyzed data from 1411 participants, recruited between 2008 and 2011, in the Maternal-Infant Research on Environmental Chemicals study. Our primary outcome was a composite inflammatory index derived by summing the z-scores of eight proinflammatory biomarkers. Using multivariable linear regression models, we quantified associations between each PFAS and the inflammatory index and individual biomarkers. We quantified the effects of the PFAS mixture using weighted quantile sum regression, and evaluated effect modification using product terms and sex-stratified models.

**Results:**

Each doubling of PFOA and PFHxS was associated with a 0.38 (95% CI, 0.09, 0.67) and 0.21 (95% CI, 0.01, 0.41) SD increase in the proinflammatory index, respectively. A one-quartile increase in the PFAS mixture was associated with a 0.40 (95% CI, 0.09, 0.71) SD increase in the proinflammatory index. In individual models, we observed positive associations between PFAS and concentrations of monocyte chemoattractant protein-1, macrophage inflammatory protein-1β, and matrix metalloproteinases-9; however, the magnitude and precision varied according to the specific PFAS. Sex-specific findings were identified in few PFAS-biomarker associations.

**Conclusions:**

PFOA, PFOS, and PFHxS, individually and as a mixture, were positively associated with proinflammatory biomarkers during pregnancy.

## Abstract 2

Supplemental Digital Content is available in the text.

**What this study adds**

We present the first identified analysis of prenatal PFAS concentrations and a comprehensive suite of inflammatory biomarkers. Specifically, we quantified associations between the first trimester concentrations of PFOA, PFOS, and PFHxS and the third trimester biomarkers of inflammation, including a derived proinflammatory index. We show that prenatal exposure to these three PFAS, both individually and as a mixture, was positively associated with a proinflammatory index, and this association was likely driven by proinflammatory chemokines, such as MCP-1 and MIP-1β. These findings provide insight into potential mechanisms underlying the adverse health effects of elevated PFAS exposure during pregnancy.

## Introduction

Per- and polyfluoroalkyl substances (PFAS) are a class of persistent environmental contaminants used in a wide range of consumer, commercial, and industrial products, and are ubiquitous in the environment.^1,2^ The highly fluorinated carbon backbone and amphipathic nature of PFAS contribute to their high chemical stability and ability to repel water and oils. Although Canada has prohibited the manufacturing, use, sale, and import of certain long-chain PFAS, including perfluorooctane sulfonate (PFOS), perfluorooctanoic acid (PFOA), and long-chain perfluorocarboxylic acids,^3^ biomonitoring studies continue to detect these legacy PFAS in over 99% of Canadians.^4^

PFAS exposure has been associated with a diverse array of adverse health outcomes,^5–7^ including dysregulated inflammatory processes, which range from both immunoenhancement to immunosuppression.^8–10^ During pregnancy, these opposing immune system processes must be highly regulated to maintain healthy maternal and birth outcomes.^11,12^ Dysregulation of maternal inflammatory profiles is associated with pregnancy complications, such as gestational diabetes and preeclampsia.^11,13^ There is emerging epidemiological evidence to suggest that PFAS may dysregulate inflammatory processes during pregnancy.^14–16^ The Maternal Adiposity, Metabolism and Stress Study (MAMAs) reported that PFOA, PFOS, and ΣPFASs were positively associated with IL-6.^14^ Additionally, authors of a nested case-control study of preterm birth in China reported positive associations between PFOS and monocyte chemoattractant protein (MCP)-1 concentrations yet inverse associations between PFOA and with interleukin (IL)-8 concentrations.^15^ However, the Spanish INfancia y Medio Ambiente Birth Cohort Study (INMA) reported no significant association between first trimester PFOA, PFOS, PFHxS, and perfluorononanoic acid (PFNA) concentrations and circulating first trimester C-reactive protein (CRP) concentrations.^16^

The association between PFAS and some pregnancy outcomes has been shown to be modified by fetal sex.^17,18^ For example, Borghese et al. reported a positive association between first trimester plasma PFOS and PFHxS concentrations and gestational hypertension in pregnant people carrying male, but not female, fetuses.^18^ Sex-specific patterns have also been seen in studies measuring circulating levels of proinflammatory cytokines during pregnancy; pregnant people carrying male fetuses tended to have higher concentrations compared with those carrying female fetuses.^19,20^ Therefore, we hypothesized that the relationship between PFAS and inflammatory biomarkers might be modified by fetal sex.

We sought to further explore the relationship between PFAS exposure and immunotoxicity during pregnancy using a diverse spectrum of 19 inflammatory biomarkers measured in participants from the Maternal-Infant Research on Environmental Chemicals (MIREC) cohort study. This panel of biomarkers includes CRP, pro- and anti-inflammatory cytokines, matrix metalloproteinases, and adhesion molecules (Table S1; http://links.lww.com/EE/A233). Our primary objective was to quantify associations between first trimester plasma concentrations of PFOA, PFOS, and PFHxS (individual and combined) and third trimester biomarkers of inflammation. Although previous studies have evaluated associations between PFAS and select inflammatory biomarkers, such as CRP, MCP-1, IL-6, IL-8, IL-10, and TNF-α, during first and second trimester of pregnancy, no identified studies have analyzed third trimester concentrations, which may be a preferable indicator of inflammatory status before delivery.^14–16^ Additionally, measurement of a broad panel of biomarkers better captures the complex interplay of pro- and anti-inflammatory processes that are critical for fetal development and perinatal health.^21,22^ Our secondary objective was to determine if the association between PFAS and inflammation during pregnancy was modified by fetal sex.

## Methods

### Study design and participants

We used data from the MIREC study, a pan-Canadian cohort study that recruited pregnant participants (n = 2001) during their first trimester from 10 cities between 2008 and 2011.^23^ Participants were eligible for inclusion if they were 18 years of age or older, <14 weeks of gestation, able to communicate in English or French, and planning to deliver at a local hospital.^23^ Participants provided demographic and lifestyle information (e.g., smoking, alcohol, and medication use) and biospecimens during study visits. The present analysis included those with live, singleton births (n = 1533) (Figure S1; http://links.lww.com/EE/A233). The original MIREC study was approved by the research ethics boards at Health Canada/Public Health Agency of Canada, Sainte Justine’s Hospital (Montreal, Quebec, Canada), and all participants provided informed consent before participating. This analysis was approved by the Health Canada’s Research Ethics Board (REB 2021-003H) and the Carleton University’s Research Ethics Board.

### First trimester plasma perfluoroalkyl substances

Maternal blood samples were collected during the first trimester clinic visit using 10-mL sterile vacutainer tubes. The mean (SD) of gestational age in weeks at the blood draw was 11.6 (1.5) weeks. Within 2 hours of the blood draw, samples were centrifuged, and plasma was aliquoted into smaller cryovials and stored at −80 °C. Analysis of PFOA, PFOS, and PFHxS concentrations was performed by the Laboratoire de Toxicologie Institut National de Santé Publique du Quebec (Quebec City, Quebec, Canada), accredited by the Standards Council of Canada. PFAS measurements were completed using ultra high-pressure liquid chromatography (ACQUITY UPLC System; Waters Corporation, Milford, Massachusetts). The limits of detection (LODs) for PFOA and PFOS were 0.1 and 0.3 μg/L, respectively. PFHxS had two LODs, 0.2 and 0.3 μg/L; we applied the more conservative LOD of 0.3 μg/L.^24^

### Third trimester plasma inflammatory biomarkers

Nineteen inflammatory biomarkers were measured in the third trimester (mean [SD] gestational age = 33.1 [1.5] weeks) plasma samples (Table 1 and Table S1; http://links.lww.com/EE/A233). Maternal whole blood was treated with ethylenediaminetetraacetic acid (EDTA) and a serine protease inhibitor, phenylmethylsulfonyl fluoride, then clarified to obtain plasma. MMPs, CRP, granulocyte-macrophage colony-stimulating factor (GM-CSF), interferon gamma (IFN-γ), IL-2, 6, 8, 12, and tumor necrosis factor alpha (TNF-α) were measured using Milliplex Map kits (Millipore, Canada). Vascular cell adhesion molecule (VCAM), intracellular adhesion molecule (ICAM), vascular endothelial growth factor (VEGF), MCP-1, and macrophage inflammatory protein-1 beta (MIP-1β) were measured using Bio-Plex Pro Human panels (Bio-Rad, Canada). Laboratory analyses were conducted by the Environmental Health Science and Research Bureau at Health Canada. Intra and interassay coefficients of variation for all biomarkers were below 11% and 20%, respectively.^25^

**Table 1.** Descriptive statistics for inflammatory biomarkers in third trimester maternal plasma samples from participants in the MIREC study (2008–2011).

Biomarker	n	LOD	%<LOD	Min	25th percentile	Median	75th percentile	Max	GM (95% CI)
MIP-1β (pg/mL)	1533	2.4	0	8.05	46.3	57.4	72.6	469	57.8 (56.72, 58.89)
MCP-1 (pg/mL)	1519	1.1	0	6.49	28.2	37.6	50.8	230	38.0 (37.11, 38.95)
TNF-α (pg/mL)	1529	0.07	0	0.2	3.24	4.30	5.83	133	4.37 (4.26, 4.48)
IFN-γ (pg/mL)	1533	0.18	0.7	<LOD	2.23	4.45	8.28	204	4.18 (3.95, 4.44)
IL-2 (pg/mL)	1532	0.26	15.6	<LOD	0.49	1.17	2.68	216	1.16 (1.09, 1.25)
IL-6 (pg/mL)	1529	0.2	0.7	<LOD	0.95	1.69	2.94	185	1.74 (1.66, 1.82)
IL-8 (pg/mL)	1529	0.05	0	0.18	1.46	1.98	2.72	40.4	2.07 (2.00, 2.13)
IL-10 (pg/mL)	1533	0.48	0.26	<LOD	14.0	20.6	31.4	721	21.10 (20.21, 21.97)
IL-12 (pg/mL)	1529	0.34	7.3	<LOD	1.05	2.15	5.09	461	2.34 (2.17, 2.50)
CRP (μg/mL)	1523	1.0 × 10^−6^	0	0.11	8.39	17.3	36.5	1600	17.3 (16.33, 18.28)
MMP-1 (ng/mL)	1533	0.003	0	0.044	0.44	0.70	1.10	16.4	0.71 (0.68, 0.73)
MMP-2 (ng/mL)	1510	0.2	0	6.10	53.4	67.0	81.8	6800	67.05 (65.44, 68.70)
MMP-7 (ng/mL)	1510	0.097	0	0.142	3.92	6.06	8.99	64.7	5.73 (5.54, 5.93)
MMP-9 (ng/mL)	1533	0.002	0	4.38	15.6	25.4	45.3	2060	28.5 (27.26, 29.74)
MMP-10 (ng/mL)	1503	0.005	0	0.021	0.19	0.26	0.35	3.43	0.26 (0.25, 0.27)
ICAM (ng/mL)	1528	0.0024	0	1.27	104	168	206	577	143 (140.07, 147.74)
VCAM (ng/mL)	1531	0.0006	0	42.3	193	265	329	712	251 (246.28, 255.33)
VEGF (pg/mL)	1532	0.2	2.8	<LOD	1.24	2.59	4.71	46.9	2.32 (2.20, 2.45)
GM-CSF (pg/mL)	1529	0.15	3.3	<LOD	0.62	1.29	2.43	219	1.23 (1.16, 1.30)

95% CI indicates 95% confidence intervals; CRP, C-reactive protein; GM, geometric mean; GM-CSF, granulocyte-macrophage colony-stimulating factor; ICAM, intracellular adhesion molecule; IFN-γ, interferon gamma; IL, interleukin; MCP-1, monocyte chemoattractant protein-1; MIP-1β, human macrophage inflammatory protein-1 beta; MMP, matrix metalloproteinase; TNF-*α*, tumor necrosis factor alpha; VCAM, vascular cell adhesion molecule; VEGF, vascular endothelial growth factor.

Our primary outcome was a composite index of proinflammatory biomarkers using knowledge of biomarker function.^25^ This index was derived by summing the standardized z-scores of MIP-1β, TNF-α, IFN-γ, MCP-1, IL-2, IL-6, IL-8, and IL-12 using the within sample means and SDs to calculate each z-score.

### Covariates

We identified covariates using a priori knowledge of associations between covariates and either inflammatory biomarkers or plasma PFAS concentrations during pregnancy.^26–29^ Covariates included in our final model were identified using a directed acyclic graph (Figure S2; http://links.lww.com/EE/A233) and included the following: maternal age, parity, education, third trimester cigarette smoking status at visit 3, prepregnancy body mass index (BMI), race and ethnicity, reported physical activity level, and gestational age at the third trimester blood collection. Participant prepregnancy BMI was obtained using self-reported prepregnancy weight and measured height and categorized according to the WHO guidelines.^30^ These variables were included in multivariable models as defined in Table S2; http://links.lww.com/EE/A233 with the exception of maternal age, which was treated as continuous variables. We performed a complete case analysis in all models; missingness for covariate data ranged from 0.13% (third trimester smoking) to 7.4% (prepregnancy BMI).

Prenatal concentrations of circulating inflammatory biomarkers may be influenced by other factors, such as regular use of anti-inflammatory medications, preeclampsia, preexisting type 1 (T1D) or type 2 diabetes (T2D), impaired glucose tolerance (IGT), or gestational diabetes mellitus (GDM). We, therefore, conducted four sensitivity analyses excluding the following participants: (1) who reported regularly taking anti-inflammatory medications at the first trimester visit (n = 54); (2) who were diagnosed with preeclampsia (n = 38); (3) who self-reported prior T1D or T2D (n = 14); or (4) IGT or GDM (n = 76). Women were asked about their history of diabetes and medication use in the baseline questionnaire. Systolic (SBP) and diastolic blood pressure (DBP) were assessed by clinical staff using a sphygmomanometer at three prenatal clinic visits (at 6–13, 14–26, and 27–40 weeks). Participants were considered as having gestational hypertension if SBP was ≥140 mmHg and/or DBP was ≥90 mmHg at a gestational age of 20 weeks or later.^31^ Preeclampsia was defined as gestational hypertension accompanied by either (a) proteinuria (defined as protein dipstick test ≥1 + OR proteinuria in 24-hour urine ≥300 mg/24 hours or ≥0.3 g/L) or (b) related maternal complications (including disseminated intravascular coagulation, pulmonary edema, convulsions-eclampsia, transfusion, elevated liver enzymes, and/or platelet count < 50 × 10^9^/L).^18,32^ IGT and GDM data were obtained by chart review. Diagnoses were based on the results of a 50 g glucose challenge test (GCT) and 75 or 100 g oral glucose tolerance test (OGTT).^33^

### Statistical analysis

We calculated descriptive statistics for exposure and outcome variables and visualized the associations between individual PFAS and outcome biomarkers using locally weighted smoothing (LOESS) plots to assess linearity. We examined Spearman correlations between PFAS and between the inflammatory biomarkers. Values below the LOD for both exposure and outcome variables were assigned a value of LOD/2. We quantified associations between PFAS and inflammatory biomarkers using multivariable linear regression models, adjusting for aforementioned covariates. Plasma concentrations of PFAS and outcome biomarkers were log_2_ transformed to normalize distributions and beta coefficients were back transformed to obtain the percent change in inflammatory biomarkers per doubling of PFAS concentrations. In the models examining PFAS and the proinflammatory index, the β coefficient is interpreted as a one-SD change, or change in the z-score, in the inflammatory index per doubling of PFAS concentrations. We conducted regression diagnostics to ensure the assumptions of linear regression were met and to ensure normality of residuals.

We used weighted quantile sum (WQS) regression to quantify the association between a PFAS mixture, comprising PFOA, PFOS, and PFHxS, and the inflammatory biomarker index using the gWQS package.^34,35^ This approach estimates the joint effect a one-quantile change in multiple exposures and calculates relative contribution (weights) of each individual exposure.^36^ We created the WQS index using quartiles of PFAS and calculated weights derived from 100 bootstrap models and a 40:60 split of training and validation datasets. We defined the mixture to have a positive association with the proinflammatory index, given our hypothesis that the mixture would be related to higher levels of inflammation. To stabilize the WQS results, we used repeated holdout validation with the same parameters as the WQS.^37^

We also explored the potential for effect modification by fetal sex by calculating the product terms and analyzing sex-stratified models. Our threshold for effect modification was a product term *P*-value <0.1.

All statistical analyses were performed using R Statistical Software (Version 1.4.1106, R Core Team, 2021). Data visualization was completed using R and GraphPad Prism (Version 9.1.1).

## Results

### Study population characteristics

Participants in our analytic sample (n = 1411) were predominantly White (84.8%), with normal prepregnancy BMI (61.1%), nonsmokers (88.5%), and over the age of 30 years (62.8%) (Table S2; http://links.lww.com/EE/A233). The three PFAS were detected in between 94.3% and 99.8% of participants (Table S3; http://links.lww.com/EE/A233). Geometric mean concentrations for first trimester plasma PFOA, PFOS, and PFHxS concentrations were 1.66, 4.57, and 1.01 µg/L, respectively (Table S3; http://links.lww.com/EE/A233). These PFAS were moderately correlated with each other: PFOA and PFOS (r = 0.56), PFOA and PFHxS (r = 0.49), PFOS and PFHxS (r = 0.54).

Twelve of the 19 inflammatory biomarkers were detected in all participants. IL-2 and IL-12 were the only biomarkers with detection rates below 95%, with 84.4% detection for IL-2 and 92.7% detection for IL-12. Geometric mean concentrations ranged from 1.16 pg/mL for IL-2 to 17.3 µg/L for CRP (Table 1). Biomarker correlation coefficients ranged from −0.20 to 0.74 and were strongest between ICAM and VCAM (Figure S3; http://links.lww.com/EE/A233). The mean (SD) Z-score for the proinflammatory index was 0.011 (4.3).

### Associations between individual perfluoroalkyl substances and perfluoroalkyl substances mixture with proinflammatory index

**Figure 1.**
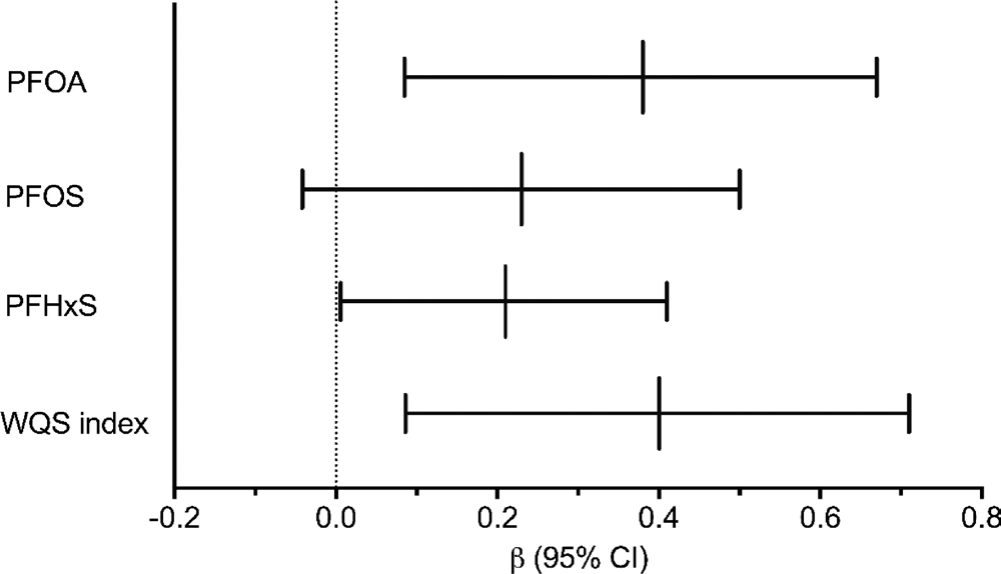
Change in the z-score of the third trimester proinflammatory index (MIP-1-β, IFN-γ, TNF-α, MCP-1, IL-2, IL-6, IL-8, IL-12) for each doubling of first trimester PFOA, PFOS, and PFHxS concentrations and for each one-quartile increase in the Weighted Quantile Sum (WQS) index of all three PFAS. Models are adjusted for maternal age, prepregnancy BMI, education, race, parity, physical activity, smoking status at visit 3, gestational age at visit 3. β indicates parameter estimate; 95% CI, 95% confidence interval.

Each doubling of PFOA, PFOS, and PFHxS was associated with a 0.38 (95% CI, 0.08, 0.67), 0.23 (95% CI, −0.042, 0.50), and 0.21 (95% CI, 0.01, 0.41) change in the z-score in the proinflammatory index, respectively (Figure 1). There were subtle differences in the precision of all three PFAS in sensitivity analysis, but overall, the direction and magnitude of effects were consistent with the primary analysis (Tables S4–S6; http://links.lww.com/EE/A233). Each one-quartile change in the PFAS WQS index was associated with a 0.40 (95% CI, 0.086, 0.71) unit increase in the proinflammatory index (Figure 1). PFHxS had the highest weight in the mixture model (0.42) followed by PFOS (0.33) and PFOA (0.26).

### Associations between individual perfluoroalkyl substances and inflammatory biomarkers

**Figure 2.**
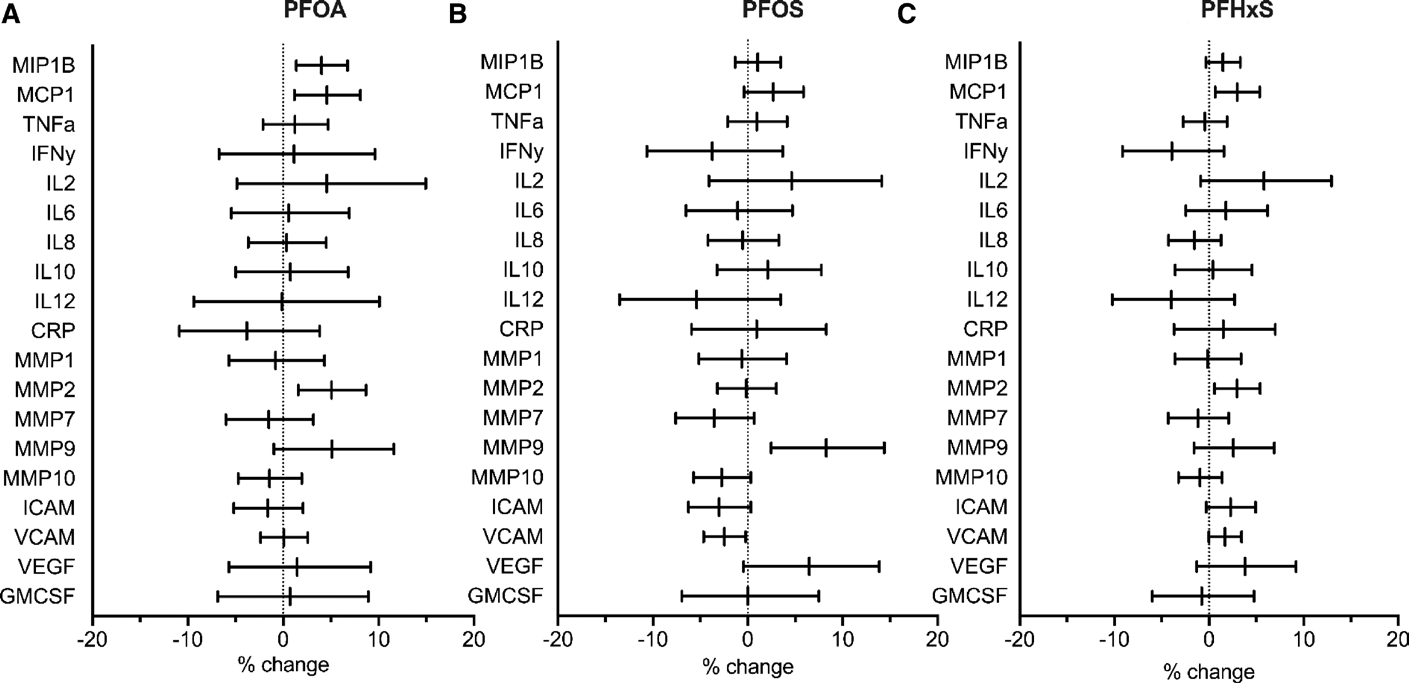
Percent change in biomarker concentrations per doubling of first trimester concentrations of perfluoroalkyl substances. (A) PFOA, (B) PFOS, or (C) PFHxS concentrations with 95% CI. Models are adjusted for maternal age, prepregnancy BMI, education, race, parity, physical activity, smoking status at visit 3, gestational age at visit 3. PFAS were measured in micrograms per liter. Biomarkers were measured in picograms per liter, and CRP was measured in nanograms per liter

We observed positive associations between PFAS and several individual proinflammatory cytokines or chemokines. Both PFOA and PFHxS were positively associated with MCP-1 (Figure 2A and 2C). Specifically, each doubling of first trimester plasma PFOA concentrations was associated with a 4.59% (95% CI, 1.20, 8.11) increase in MCP-1, while each doubling of PFHxS was associated with a 2.98% (95% CI, 0.65, 5.36) increase in MCP-1 (Figure 2A and 2C). We also observed a positive association between PFOA and MIP-1β (4.04% [95% CI, 1.40, 6.75]; Figure 2A). PFAS concentrations were generally not associated with interleukins, TNF-α, IFN-γ, or CRP (Figure 2).

We observed positive associations between PFAS and individual MMPs. PFOA and PFHxS were associated with 5.08% (95% CI, 1.59, 8.69) and 2.98% (95% CI, 0.59, 5.42) higher MMP-2 concentrations, respectively (Figure 2A and 2C). Seven participants had elevated plasma MMP-2 concentrations in the range of 4557.75 to 6803.10 ng/mL. Excluding these 7 participants attenuated the association between PFOA and MMP-2 (3.60% [95% CI, 0.98, 6.29]) and between PFHxS and MMP-2 (2.83% [95% CI, 1.01, 4.68]). Each doubling of PFOS was also associated with an 8.27% (95% CI, 2.48, 14.39) increase in MMP-9 concentrations (Figure 2B).

Mixed associations were observed between PFAS and biomarkers of vascular inflammation. PFOS was inversely associated with VCAM (−2.64 [95% CI, −4.65, −0.23]) and ICAM (−3.02 [95% CI, −6.25, 0.33]) (Figure 2B), while the direction of association between PFHxS and VCAM and ICAM was positive (Figure 2C). The direction and magnitude of observed associations in all sensitivity analyses were largely consistent with that of the analytical sample (Tables S4–S6; http://links.lww.com/EE/A233).

### Effect modification by fetal sex on individual perfluoroalkyl substances and outcome biomarker associations

We observed effect modification (product term *P* < 0.1) by fetal sex in 8 models examining individual PFAS and inflammatory biomarkers. Among these models, associations between PFAS concentrations and inflammatory biomarkers tended to be positive or null in participants carrying male fetuses and inverse in participants carrying female fetuses, with the exception of MMP-9 and IL-6 (Table S7; http://links.lww.com/EE/A233). For example, PFHxS was positively associated with IL-10 in participants carrying male fetuses (6.91% [95% CI, 1.00, 13.16]) and inversely in participants carrying female fetuses (−5.68% [95% CI, −11.00, −0.04]) (product term *P* < 0.01). We did not observe any evidence of sex-specific differences in models evaluating the proinflammatory index (Table S7; http://links.lww.com/EE/A233).

## Discussion

In this Canadian pregnancy cohort, plasma concentrations of three PFAS, both individually and as a mixture, during early pregnancy were positively associated with third trimester proinflammatory biomarker concentrations. Each PFAS was positively associated with the proinflammatory index with the strongest magnitude of association observed for PFOA. The positive associations between PFAS and the proinflammatory index may be driven by chemokines such as MCP-1 and MIP-1β as we observed evidence of associations with these biomarkers in the individual models.

Our observed positive associations between PFAS and both MCP-1 and MIP-1β are of interest because elevated levels of these chemokines may be associated with adverse pregnancy outcomes. MCP-1 and MIP-1β are proinflammatory chemokines responsible for recruiting monocytes and other leukocytes to sites of inflammation.^38^ Increased third trimester MCP-1 concentrations have been associated with preterm birth, small for gestational age, and infections during pregnancy.^25,39^ Similarly, increased MIP-1β concentrations during pregnancy has been associated with spontaneous preterm delivery.^40^ While the individual biomarkers suggest potential for adverse clinical outcomes, our derived composite proinflammatory index, which is reflective of overall systemic inflammation, has not been examined in relation to any specific clinical outcome. No identified studies have evaluated whether these biomarkers mediate associations between PFAS and pregnancy complications.

We identified parallels between our findings and other published studies that evaluated associations between PFAS and inflammatory biomarkers.^14–16^ Consistent with our results, Matilla-Santander et al. reported null associations between first trimester PFAS (PFOA, PFOS, PFHxS, and PFNA) and concurrently measured CRP concentrations in the INMA study.^16^ MIREC participants had, on average, lower plasma PFOA and PFOS levels, and higher PFHxS levels than participants in INMA cohort; these differences may be partially due to the differing windows of recruitment for the two studies (MIREC: 2008–2011; INMA: 2003–2008). Third trimester median CRP levels in MIREC participants were approximately four-fold higher than the first trimester CRP levels in INMA study participants. This difference may be partially attributed to the increase in CRP levels throughout pregnancy.^41^ The INMA study did not measure any of the other biomarkers included in our analysis.^16^ Also consistent with our findings, authors of the Shanxi Province (China) nested case-control study reported positive associations between PFOS and MCP-1 concentrations measured between 4 and 22 weeks gestation in participants recruited between 2009 and 2013.^15^ In contrast to our findings, PFOA was inversely associated with IL-8 in the Shanxi Province nested case-control study. MIREC participants had two- to three-fold higher median PFOA and PFOS concentrations compared with the Shanxi Province nested case-control study. PFOA and PFOS concentrations measured in the Shanxi Province nested case-control study were also lower than what was observed in other studies measuring plasma PFAS during a similar time period in pregnant women in China.^42,43^ MCP-1 and IL-8 plasma concentrations were approximately four-fold and four-fold lower in the MIREC study compared with the Shanxi Province nested case-control study, respectively. Also in contrast to our findings, the MAMAs study of San Francisco, California reported positive associations between PFOA, PFOS, and ΣPFASs with longitudinal IL-6 concentrations measured in the second trimester, and 3- and 9-months postpartum.^14^ MIREC and MAMAs participants had similar geometric mean concentrations of PFOA but MIREC participants had approximately 1.5-fold higher PFOS concentrations. Additionally, MIREC participants had approximately three-fold higher median IL-6 concentrations in the third trimester compared with the second trimester concentrations reported in the MAMAs study. In addition to these differences, the MAMAs cohort included women of overweight and obese prepregnancy BMI. Discrepancies between MIREC and MAMAs could be due to the composition of the study population or timing of biomarker measurements.^14^ Inflammatory biomarker levels tend to increase during the first trimester promoting angiogenesis and blastocyst implantation.^44^ Concentrations of interleukins and other cytokines such as TNF-α tend to decrease in the second trimester during key periods of fetal growth and increase again during the third trimester as parturition nears.^44,45^ Therefore, it is difficult to directly compare our findings of third trimester biomarker concentrations to those studies that measured biomarkers either earlier in pregnancy or postpartum.

The biological plausibility of our findings is supported by evidence from rodent studies demonstrating that PFAS exposure elicits immunomodulatory and immunotoxic effects in vivo, including upregulation of proinflammatory genes and increased circulating inflammatory biomarkers, such as IL-6 and IL-1β.^46–50^ As there are multiple possible molecular targets for PFAS, disentangling a specific signaling pathway responsible for dysregulated inflammation is challenging. One potential pathway is through activation of nuclear receptors known as peroxisome proliferator-activated receptors (PPARs), which are canonically activated by fatty acids and highly expressed in the placenta.^51^ PPARs are involved in regulating fatty acid disposition, metabolism, and the inflammatory response.^51,52^ There is mounting evidence for an interaction between exposure to PFAS and placental PPAR signaling, which may mediate associations between these chemicals and adverse pregnancy outcomes.^9^ Other relevant pathways include activation of nuclear receptors such as the constitutive androgen receptor (CAR) and pregame X receptor (PXR), which are involved in hepatic cell proliferation and other intermediary metabolic processes, including inflammation.^53^

Our sex-specific findings are consistent with observed effect modification by sex in the analysis of PFAS and preeclampsia in MIREC participants.^18^ We noted particularly compelling effect modification by fetal sex for the association between PFHxS with IL-10, a known anti-inflammatory biomarker (Table S7; http://links.lww.com/EE/A233).^54^ The mechanisms underlying fetal sex-specific differences in maternal inflammatory profiles are unclear. Some studies report that individuals carrying male fetuses exhibit increased levels of proinflammatory biomarkers^20^ and lower levels of anti-inflammatory biomarkers.^19^ However, our study and others reported no or minimal differences in biomarker concentrations between fetal sex (Table S8; http://links.lww.com/EE/A233).^55^ While our results showed that fetal sex modifies the association between PFAS and a small subset of inflammatory biomarkers, these findings will need to be replicated in other populations. Our study is the first identified analyses of these associations. It is possible the observed findings were due to chance and worth noting that effect modification was not observed with our primary outcome or in the majority of individual models.

MIREC is a prospective, multisite pan-Canadian cohort study uniquely suited to investigate associations between exposure to environmental contaminants and maternal/fetal health outcomes. Due to the extensive repository of data available from the MIREC study, we were able to control for key confounders. The longitudinal study design provides temporality between plasma PFAS measurements and inflammatory biomarkers. Additionally, we minimized potential misclassification of PFAS by using first trimester plasma measurements that precede the onset of key physiological changes during pregnancy, such as increased glomerular filtration rate and plasma volume expansion.^56,57^ To our knowledge, this is the first study examining first trimester plasma concentrations of PFAS, both individually and as a mixture, and third trimester plasma concentrations of an extensive panel of inflammatory biomarkers. Investigating an extensive panel of biomarkers allowed for a comprehensive analysis of the inflammatory profile of MIREC participants during pregnancy. Future research can build upon this work by evaluating potential mediation between PFAS, inflammatory biomarkers, and clinical outcomes.

Our study has four potential limitations. First, because of data availability, we relied on inflammatory biomarker concentrations at a single-time point and were not able to account for the natural fluctuations that arise from the changing fetal demands and physiological stress of pregnancy.^45^ Second, we recognize that inflammatory biomarker protein concentrations may not be indicative of biomarker activity, particularly for MMPs.^58^ Nevertheless, despite these limitations, third trimester measurements of these biomarkers provide clinically meaningful indicators of potential adverse birth outcomes as they are proximal to delivery.^45^ Third, owing to the large number of associations examined, observed statistical associations for the individual PFAS and biomarkers, but not the proinflammatory index, are subject to Type 1 error. We did not adjust for multiple comparisons as this would impede our ability to identify possible associations and thus increase our risk of Type 2 error.^59^ Fourth, although our analysis excluded participants without measured inflammatory biomarkers, any resulting potential bias is likely minimal; outcome data are available for approximately 85% of the participants with first trimester PFAS data and PFAS concentrations were comparable between those with and without outcome biomarker data. Moreover, differences in sociodemographic characteristics between these groups are unlikely to be differentially related to exposures and outcomes (Table S9; http://links.lww.com/EE/A233).

In conclusion, we provide evidence that first trimester maternal PFAS concentrations, both individually and as a mixture, are positively associated with third trimester proinflammatory biomarker concentrations. Future studies are necessary to examine a wider suite of PFAS analytes and examine whether these inflammatory biomarkers are on the causal pathway between PFAS and pregnancy and child complications.

## ACKNOWLEDGMENTS

The authors are grateful to the MIREC families for their interest and participation and to the dedicated site and coordinating center staff for recruiting the participants and collecting and managing the data and biospecimens. The MIREC study was funded by Health Canada’s Chemicals Management Plan. We also thank Linda Dodds for her roles in the MIREC study design and development of derived variables for IGT/GDM.

## Conflicts of interest statement

The authors declare that they have no conflicts of interest with regard to the content of this report.

The MIREC Study was supported by Health Canada’s Chemicals Management Plan at Health Canada, the Ontario Ministry of the Environment, and the Canadian Institutes for Health Research (grant MOP-81285). This study was also supported by a 2022 Carleton University Research Achievement Award.

Data ussed in this analysis are confidential and not available for distribution. Some analytical code may be provided upon request.

## Supplementary Material

## References

[1] Glüge J Scheringer M Cousins IT. An overview of the uses of per- and polyfluoroalkyl substances (PFAS). Environ Sci Process Impacts. 2020;22:2345–2373. 33125022 10.1039/d0em00291g PMC7784712

[2] Buck RC Franklin J Berger U. Perfluoroalkyl and polyfluoroalkyl substances in the environment: terminology, classification, and origins. Integr Environ Assess Manag. 2011;7:513–541. 21793199 10.1002/ieam.258 PMC3214619

[3] Government of Canada. Toxic Substances List: PFOS. Published online 2018. Available at: https://www.canada.ca/en/environment-climate-change/services/management-toxic-substances/list-canadian-environmental-protection-act/perfluorooctane-sulfonate.html

[4] Health Canada. Sixth Report on Human Biomonitoring of Environmental Chemicals in Canada. 2021.Available at: www.canada.ca/en/health-canada/services/environmental-workplacehealth/reports-publications/environmental-contaminants/sixth-report-human-biomonitoring.html

[5] Lau C Anitole K Hodes C Lai D Pfahles-Hutchens A Seed J. Perfluoroalkyl acids: a review of monitoring and toxicological findings. Toxicol Sci. 2007;99:366–394. 17519394 10.1093/toxsci/kfm128

[6] Andersen ME Butenhoff JL Chang SC. Perfluoroalkyl acids and related chemistries - Toxicokinetics and modes of action. Toxicol Sci. 2008;102:3–14. 18003598 10.1093/toxsci/kfm270

[7] Fenton SE Ducatman A Boobis A. Per- and Polyfluoroalkyl substance toxicity and human health review: current state of knowledge and strategies for informing future research. Environ Toxicol Chem. 2021;40:606–630. 33017053 10.1002/etc.4890 PMC7906952

[8] DeWitt JC Blossom SJ Schaider LA. Exposure to per-fluoroalkyl and polyfluoroalkyl substances leads to immunotoxicity: epidemiological and toxicological evidence. J Expo Sci Environ Epidemiol. 2019;29:148–156. 30482935 10.1038/s41370-018-0097-y PMC6380927

[9] Szilagyi JT Avula V Fry RC. Perfluoroalkyl substances (PFAS) and their effects on the placenta, pregnancy, and child development: a potential mechanistic role for placental peroxisome proliferator–activated receptors (PPARs). Curr Environ Health Rep. 2020;7:222–230. 32812200 10.1007/s40572-020-00279-0 PMC7473499

[10] Fairley KJ Purdy R Kearns S Anderson SE Meade BJ. Exposure to the immunosuppresant, perfluorooctanoic acid, enhances the murine IgE and airway hyperreactivity response to ovalbumin. Toxicol Sci. 2007;97:375–383. 17369199 10.1093/toxsci/kfm053

[11] Pantham P Aye ILMH Powell TL. Inflammation in maternal obesity and gestational diabetes mellitus. Placenta. 2015;36:709–715. 25972077 10.1016/j.placenta.2015.04.006 PMC4466145

[12] deRosset L Strutz KL. Developmental origins of chronic inflammation: a review of the relationship between birth weight and C-reactive protein. Ann Epidemiol. 2015;25:539–543. 25726300 10.1016/j.annepidem.2015.01.003

[13] Harmon AC Cornelius DC Amaral LM. The role of inflammation in the pathology of preeclampsia. Clin Sci. 2016;130:409–419. 10.1042/CS20150702 PMC5484393 26846579

[14] Zota AR Geller RJ Romano LE. Association between persistent endocrine-disrupting chemicals (PBDEs, OH-PBDEs, PCBs, and PFASs) and biomarkers of inflammation and cellular aging during pregnancy and postpartum. Environ Int. 2018;115:9–20. 29533840 10.1016/j.envint.2018.02.044 PMC5970048

[15] Liu X Chen D Wang B. Does low maternal exposure to per- And polyfluoroalkyl substances elevate the risk of spontaneous preterm birth? a nested case-control Study in China. Environ Sci Technol. 2020;54:8259–8268. 32510220 10.1021/acs.est.0c01930

[16] Matilla-Santander N Valvi D Lopez-Espinosa MJ. Exposure to perfluoroalkyl substances and metabolic outcomes in pregnant women: evidence from the Spanish INMA birth cohorts. Environ Health Perspect. 2017;125:117004–117004. 29135438 10.1289/EHP1062 PMC5947948

[17] Yang L Ji H Liang H. Associations of perfluoroalkyl and polyfluoroalkyl substances with gestational hypertension and blood pressure during pregnancy: a cohort study. Environ Res. 2022;215:114284–114284. 36088993 10.1016/j.envres.2022.114284

[18] Borghese MM Walker M Helewa ME Fraser WD Arbuckle TE. Association of perfluoroalkyl substances with gestational hypertension and preeclampsia in the MIREC study. Environ Int. 2020;141. doi:10.1016/j.envint.2020.105789. 10.1016/j.envint.2020.105789 32408216

[19] Ramiro-Cortijo D de la Calle M Böger R. Male fetal sex is associated with low maternal plasma anti-inflammatory cytokine profile in the first trimester of healthy pregnancies. Cytokine. 2020;136. doi:10.1016/j.cyto.2020. 10.1016/j.cyto.2020.155290 32956948

[20] Enninga EAL Nevala WK Creedon DJ Markovic SN Holtan SG. Fetal sex-based differences in maternal hormones, angiogenic factors, and immune mediators during pregnancy and the postpartum period. Am J Reprod Immunol. 2015;73:251–262. 25091957 10.1111/aji.12303 PMC4317383

[21] Aluvihare VR Kallikourdis M Betz AG. Regulatory T cells mediate maternal tolerance to the fetus. Nat Immunol. 2004;5:266–271. 14758358 10.1038/ni1037

[22] Hanna J Goldman-Wohl D Hamani Y. Decidual NK cells regulate key developmental processes at the human fetal-maternal interface. Nat Med. 2006;12:1065–1074. 16892062 10.1038/nm1452

[23] Arbuckle TE Fraser WD Fisher M. Cohort profile: the maternal-infant research on environmental chemicals research platform. Paediatr Perinat Epidemiol. 2013;27:415–425. 23772943 10.1111/ppe.12061

[24] Fisher M Arbuckle TE Liang CL. Concentrations of persistent organic pollutants in maternal and cord blood from the maternal-infant research on environmental chemicals (MIREC) cohort study. Environ Health. 2016;15:1–14. 27142700 10.1186/s12940-016-0143-y PMC4855498

[25] Kumarathasan P Williams G Bielecki A. Characterization of maternal plasma biomarkers associated with delivery of small and large for gestational age infants in the MIREC study cohort. PLoS One. 2018;13:e0204863–e0204817. 30383759 10.1371/journal.pone.0204863 PMC6211634

[26] Berg V Nøst TH Huber S. Maternal serum concentrations of per- and polyfluoroalkyl substances and their predictors in years with reduced production and use. Environ Int. 2014;69:58–66. 24815340 10.1016/j.envint.2014.04.010

[27] Lee J Taneja V Vassallo R. Cigarette smoking and inflammation: cellular and molecular mechanisms. J Dent Res. 2012;91:142–149. 21876032 10.1177/0022034511421200 PMC3261116

[28] Friedman EM Herd P. Income, education, and inflammation: differential associations in a national probability sample (the midus study). Psychosom Med. 2010;72:290–300. 20100883 10.1097/PSY.0b013e3181cfe4c2 PMC2855758

[29] Cerqueira E Marinho DA Neiva HP Lourenço O. Inflammatory effects of high and moderate intensity exercise—a systematic review. Front Physiol. 2020;10. doi:10.3389/fphys.2019.01550. 10.3389/fphys.2019.01550 PMC6962351 31992987

[30] World Health Organization. A Healthy Lifestyle - WHO Recommendations. 2010. Available at: https://www.who.int/europe/news-room/fact-sheets/item/a-healthy-lifestyle---who-recommendations

[31] Magee LA Pels A Helewa M Rey E von Dadelszen P; Canadian Hypertensive Disorders of Pregnancy (HDP) Working Group. Diagnosis, evaluation, and management of the hypertensive disorders of pregnancy. Pregnancy Hypertens. 2014;4:105–145. 26104418 10.1016/j.preghy.2014.01.003

[32] Butalia S Audibert F Côté AM; Hypertension Canada. Hypertension Canada’s 2018 guidelines for the management of hypertension in pregnancy. Can J Cardiol. 2018;34:526–531. 29731014 10.1016/j.cjca.2018.02.021

[33] Shapiro GD Dodds L Arbuckle TE. Exposure to phthalates, bisphenol a and metals in pregnancy and the association with impaired glucose tolerance and gestational diabetes mellitus: the MIREC study. Environ Int. 2015;83:63–71. 26101084 10.1016/j.envint.2015.05.016

[34] Renzetti S Paul C Allan C Ghalib B Chris G. gWQS: Generalized weighted quantile sum regression. Available at: https://CRAN.R-project.org/package=gWQS

[35] Carrico C Gennings C Wheeler DC Factor-Litvak P. Characterization of weighted quantile sum regression for highly correlated data in a risk analysis setting. J Agric Biol Environ Stat. 2015;20:100–120. 30505142 10.1007/s13253-014-0180-3 PMC6261506

[36] Zhang Y Dong T Hu W. Association between exposure to a mixture of phenols, pesticides, and phthalates and obesity: comparison of three statistical models. Environ Int. 2019;123:325–336. 30557812 10.1016/j.envint.2018.11.076

[37] Tanner EM Bornehag CG Gennings C. Repeated holdout validation for weighted quantile sum regression. MethodsX. 2019;6:2855–2860. 31871919 10.1016/j.mex.2019.11.008 PMC6911906

[38] Singh S Anshita D Ravichandiran V. MCP-1: Function, regulation, and involvement in disease. Int Immunopharmacol. 2021;101. doi:10.1016/j.intimp.2021.107598. 10.1016/j.intimp.2021.107598 PMC8135227 34233864

[39] Sean Esplin M Peltier MR Hamblin S. Monocyte chemotactic protein-1 expression is increased in human gestational tissues during term and preterm labor. Placenta. 2005;26:661–671. 16085045 10.1016/j.placenta.2004.09.012

[40] Lee SM Park KH Jung EY Cho SH Ryu A. Prediction of spontaneous preterm birth in women with cervical insufficiency: comprehensive analysis of multiple proteins in amniotic fluid. J Obstet Gynaecol Res. 2016;42:776–783. 26990253 10.1111/jog.12976

[41] Wirestam L Pihl S Saleh M Wetterö J Sjöwall C. Plasma C-Reactive protein and pentraxin-3 feference intervals during normal pregnancy. Front Immunol. 2021;12:722118. 34408755 10.3389/fimmu.2021.722118 PMC8366313

[42] Tian Y Zhou Y Miao M. Determinants of plasma concentrations of perfluoroalkyl and polyfluoroalkyl substances in pregnant women from a birth cohort in Shanghai, China. Environ Int. 2018;119:165–173. 29958117 10.1016/j.envint.2018.06.015

[43] Liu J Gao X Wang Y. Profiling of emerging and legacy per-/polyfluoroalkyl substances in serum among pregnant women in China. Environ Pollut. 2021;271:116376. 33383424 10.1016/j.envpol.2020.116376

[44] Mor G Aldo P Alvero AB. The unique immunological and microbial aspects of pregnancy. Nat Rev Immunol. 2017;17:469–482. 28627518 10.1038/nri.2017.64

[45] Jarmund AH Giskeødegård GF Ryssdal M. Cytokine patterns in maternal serum from first trimester to term and beyond. Front Immunol. 2021;12. doi:10.3389/fimmu.2021.752660. 10.3389/fimmu.2021.752660 PMC8552528 34721426

[46] Dong GH Zhang YH Zheng L Liang ZF Jin YH He QC. Subchronic effects of perfluorooctanesulfonate exposure on inflammation in adult male C57BL/6 mice. Environ Toxicol. 2012;27:285–296. 20737580 10.1002/tox.20642

[47] Qazi MR Bogdanska J Butenhoff JL Nelson BD DePierre JW Abedi-Valugerdi M. High-dose, short-term exposure of mice to perfluorooctanesulfonate (PFOS) or perfluorooctanoate (PFOA) affects the number of circulating neutrophils differently, but enhances the inflammatory responses of macrophages to lipopolysaccharide (LPS) in a similar fashion. Toxicology. 2009;262:207–214. 19540903 10.1016/j.tox.2009.06.010

[48] Pan Z Yuan X Tu W Fu Z Jin Y. Subchronic exposure of environmentally relevant concentrations of F-53B in mice resulted in gut barrier dysfunction and colonic inflammation in a sex-independent manner. Environ Pollut. 2019;253:268–277. 31319243 10.1016/j.envpol.2019.07.021

[49] Keil DE Mehlmann T Butterworth L Peden-adams MM. Gestational exposure to perfluorooctane sulfonate suppresses immune function in B6C3F1 mice. Toxicol Sci. 2008;103:77–85. 18252804 10.1093/toxsci/kfn015

[50] Lefebvre DE Curran I Armstrong C. Immunomodulatory effects of dietary potassium perfluorooctane sulfonate (PFOS) exposure in adult Sprague-Dawley rats. J Toxicol Environ Health A. 2008;71:1516–1525. 18923994 10.1080/15287390802391943

[51] Fournier T Tsatsaris V Handschuh K Evain-Brion D. PPARs and the Placenta. Placenta. 2007;28:65–76. 16834993 10.1016/j.placenta.2006.04.009

[52] Bensinger SJ Tontonoz P. Integration of metabolism and inflammation by lipid-activated nuclear receptors. Nature. 2008;454:470–477. 18650918 10.1038/nature07202

[53] Hernandez J Mota L Baldwin W. Activation of CAR and PXR by dietary, environmental and occupational chemicals alters drug metabolism, intermediary metabolism, and cell proliferation. Curr Pharmacogenomics Pers Med. 2012;7:81–105. 10.2174/187569209788654005 PMC2944248 20871735

[54] Iyer SS Cheng G. Role of Interleukin 10 transcriptional regulation in inflammation and autoimmune disease. Crit Rev Immunol. 2012;32:23–63. 22428854 10.1615/critrevimmunol.v32.i1.30 PMC3410706

[55] Mitchell AM Palettas M Christian LM. Fetal sex is associated with maternal stimulated cytokine production, but not serum cytokine levels, in human pregnancy. Brain Behav Immun. 2017;60:32–37. 27375004 10.1016/j.bbi.2016.06.015 PMC5558889

[56] Hussein W Lafayette RA. Renal function in normal and disordered pregnancy. Curr Opin Nephrol Hypertens. 2014;23:46–53. 24247824 10.1097/01.mnh.0000436545.94132.52 PMC4117802

[57] Aguree S Gernand AD. Plasma volume expansion across healthy pregnancy: a systematic review and meta-analysis of longitudinal studies. BMC Pregnancy Childbirth. 2019;19. doi:10.1186/s12884-019-2619-6. 10.1186/s12884-019-2619-6 PMC6924087 31856759

[58] Islam MS Mohanto NC Karim MR. Elevated concentrations of serum matrix metalloproteinase-2 and -9 and their associations with circulating markers of cardiovascular diseases in chronic arsenic-exposed individuals. Environ Health. 2015;14:92. 26637202 10.1186/s12940-015-0079-7 PMC4670511

[59] Rothman KJ. No adjustments are needed for multiple comparisons. Epidemiology. 1990;1:43–46. 2081237

