# Supplementary material for: Prenatal exposure to perfluoroalkyl substances and inflammatory biomarker concentrations
Source: Environ Epidemiol. 2023 Jul 25;7(4):e262. doi: 10.1097/EE9.0000000000000262 (PMC10403040; doi:10.1097/EE9.0000000000000262)
Supplement: Supplementary file 1 [file ee9-7-e262-s001.docx]

**SUPPLEMENTARY TABLES**

Table S1. Inflammatory biomarkers categorized by broad function measured in participants from the MIREC Study (2008-2011)

| **Function/Class** | **Biomarkers** |
| --- | --- |
| Pro-inflammatory | Interleukin (IL)-2, IL-6, IL-8, IL-12  Monocyte chemoattractant protein (MCP)-1  macrophage inflammatory protein-1 beta (MIP-1β)  Interferon gamma (IFN-γ)  Tumor necrosis factor-alpha (TNF-α) |
| Pro-inflammatory (acute) | C-reactive protein (CRP) |
| Vascular | Vascular cell adhesion molecule (VCAM)  Intracellular adhesion molecule (ICAM)  Vascular endothelial growth factor (VEGF) |
| Matrix Metalloproteinases (MMPs) | MMP-1, MMP-2, MMP-7, MMP-9, MMP-10 |
| Anti-inflammatory | IL-10  Granulocyte-macrophage colony-stimulating factor (GM-CSF) |

Table S2. Sociodemographic characteristics of participants (n = 1533) from the MIREC Study (2008-2011)

|  | **n (%)** |
| --- | --- |
| **Maternal age** |  |
| <24 | 97 (6.3) |
| 25-29 | 371 (24.2) |
| 30-34 | 547 (34.7) |
| 35+ | 518 (33.8) |
| **Race and ethnicity** |  |
| White | 1293 (84.3) |
| Other | 240 (15.7) |
| **Parity** |  |
| 0 | 678 (44.2) |
| 1 | 619 (40.4) |
| 2+ | 236 (15.4) |
| **Pre-pregnancy BMI** |  |
| $<$25 | 903 (58.9) |
| 25 to$<$30 | 301 (19.6) |
| $\geq$30 | 216 (14.1) |
| Missing | 113 (7.4) |
| **Income ($CAD)** |  |
| less than 50 000 | 252 (16.4) |
| 50 000 - 100 000 | 625 (40.8) |
| more than 100 000 | 594 (38.8) |
| Missing | 62 (4.0) |
| **Education** |  |
| High school diploma or less | 125 (8.2) |
| College classes or trade school | 441 (28.8) |
| Undergraduate degree | 567 (37.0) |
| Graduate school | 398 (26.0) |
| Missing | 2 (0.13) |
| **Activity Level (min/day)** |  |
| Low activity (< 30) | 137 (8.9) |
| Moderate activity (30–60) | 627 (40.9) |
| High activity (> 60) | 766 (50.0) |
| Missing | 3 (0.20) |
| **Smoking status (Visit 3)** |  |
| Never | 943 (61.5) |
| Quit during pregnancy | 111 (7.2) |
| Current | 72 (4.7) |
| Former | 405 (26.4) |
| Missing | 2 (0.13) |

Table S3. Descriptive statistics for 1^st^ trimester plasma PFAS concentrations from participants (n = 1411) in the MIREC study (2009-2011) (μg/L)

| **Contaminant** |  | **LOD** | **<LOD (%)** | **Min** | **25^th^ Percentile** | **Median** | **75^th^ Percentile** | **Max** | **Geometric mean** |
| --- | --- | --- | --- | --- | --- | --- | --- | --- | --- |
| PFOA |  | 0.1 | 0.2 | <LOD | 1.2 | 1.7 | 2.4 | 16 | 1.67 |
| PFOS |  | 0.3 | 0.2 | <LOD | 3.3 | 4.6 | 6.8 | 36 | 4.57 |
| PFHxS |  | 0.3 | 5.7 | <LOD | 0.7 | 1.0 | 1.6 | 25 | 1.01 |

Table S4. Percent change (95% CI) or β (95% CI) in inflammatory biomarker concentrations per doubling of PFOA. Highlighted boxes signify p<0.05. β can be interpreted as standard deviation-change in the index for each doubling of PFOA. Models are adjusted for maternal age, pre-pregnancy BMI, education, race, parity, physical activity, smoking status at visit 3, gestational age at visit 3*.*

| **Biomarker** | **Main analyses (n = 1411)** | **Excluding participants (n=54) who regularly take anti-inflammatory medications** | **Excluding participants (n=38) with pre-eclampsia** | **Excluding participants (n=14) with prior T1D or T2D** | **Excluding participants (n=76) with prior IGT or GDM** |
| --- | --- | --- | --- | --- | --- |
| **Percent change (95% CI)** | | | | | |
| MIP-1ß | **4.04 (1.40, 6.75)** | **4.31 (1.61, 7.09)** | **4.12 (1.48, 6.84)** | **4.10 (1.42, 6.84)** | **4.12 (1.40, 6.9)** |
| MCP-1 | **4.59 (1.20, 8.11)** | **4.00 (0.53, 7.59)** | **5.24 (1.79, 8.80)** | **4.38 (0.95, 7.93)** | **4.63 (1.15, 8.23)** |
| TNF-α | 1.24 (-2.11, 4.72) | 1.15 (-2.25, 4.67) | 1.29 (-2.09, 4.79) | 1.61 (-1.79, 5.13) | 0.58 (-2.82, 4.11) |
| IFN-γ | 1.14 (-6.7, 9.64) | 1.81 (-6.22, 10.54) | 1.28 (-6.65, 9.89) | 1.38 (-6.57, 10.01) | 1.02 (-6.9, 9.61) |
| IL-2 | 4.58 (-4.85, 14.94) | 4.67 (-4.91, 15.2) | 5.79 (-3.89, 16.43) | 5.47 (-4.13, 16.04) | 4.15 (-5.43, 14.7) |
| IL-6 | 0.57 (-5.43, 6.94) | 0.6 (-5.47, 7.04) | 0.81 (-5.28, 7.29) | 0.9 (-5.19, 7.38) | 0.9 (-5.28, 7.48) |
| IL-8 | 0.35 (-3.65, 4.53) | 0.42 (-3.66, 4.68) | 0.43 (-3.61, 4.64) | 0.67 (-3.39, 4.91) | -0.12 (-4.17, 4.11) |
| IL-10 | 0.76 (-4.97, 6.83) | 1.22 (-4.64, 7.45) | 0.45 (-5.32, 6.57) | 0.63 (-5.15, 6.78) | 1.13 (-4.70, 7.30) |
| IL-12 | -0.11 (-9.36, 10.08) | -0.51 (-9.94, 9.9) | 0.17 (-9.16, 10.47) | -0.25 (-9.58, 10.05) | -0.33 (-9.67, 9.97) |
| CRP | -3.8 (-10.87, 3.83) | -3.43 (-10.65, 4.38) | -2.36 (-9.58, 5.42) | -3.57 (-10.7, 4.14) | -3.66 (-10.79, 4.05) |
| MMP-1 | -0.79 (-5.67, 4.35) | -0.73 (-5.7, 4.5) | -0.87 (-5.75, 4.27) | -0.37 (-5.32, 4.85) | -0.97 (-5.92, 4.25) |
| MMP-2 | **5.08 (1.59, 8.69)** | **5.33 (1.77, 9.01)** | **5.09 (1.55, 8.76)** | **5.2 (1.68, 8.84)** | **5.05 (1.45, 8.78)** |
| MMP-7 | -1.51 (-6, 3.18) | -1.5 (-6.05, 3.28) | -1.9 (-6.39, 2.8) | -1.59 (-6.11, 3.15) | -2.44 (-6.96, 2.31) |
| MMP-9 | 5.13 (-0.98, 11.61) | 5.84 (-0.46, 12.53) | 5.34 (-0.87, 11.95) | 5.65 (-0.56, 12.24) | 4.26 (-1.94, 10.86) |
| MMP-10 | -1.42 (-4.69, 1.95) | -1.55 (-4.89, 1.9) | -1.21 (-4.49, 2.2) | -1.62 (-4.91, 1.79) | -1.53 (-4.84, 1.9) |
| ICAM | -1.61 (-5.17, 2.09) | -2.03 (-5.66, 1.75) | -1.39 (-4.99, 2.35) | -1.64 (-5.24, 2.10) | -1.27 (-4.86, 2.44) |
| VCAM | 0.08 (-2.36, 2.58) | 0.09 (-2.39, 2.63) | 0.06 (-2.40, 2.59) | 0.12 (-2.34, 2.65) | 0.18 (-2.31, 2.74) |
| VEGF | 1.48 (-5.67, 9.17) | 1.13 (-6.16, 8.99) | 1.89 (-5.29, 9.61) | 1.31 (-5.91, 9.09) | 3.44 (-4.01, 11.47) |
| GMCSF | 0.72 (-6.85, 8.92) | 0.99 (-6.65, 9.26) | 0.89 (-6.79, 9.21) | 1.11 (-6.57, 9.41) | 0.65 (-7.06, 8.99) |
| **β (95% CI)^1^** | | | | |  |
| Pro-inflammatory index | **0.38 (0.08, 0.67)** | **0.36 (0.06, 0.67)** | **0.41 (0.11, 0.71)** | **0.39 (0.09, 0.68)** | **0.36 (0.06, 0.66)** |

β – parameter estimate; 95% CI – 95% confidence interval; T1D – Type 1 diabetes; T2D – Type 2 diabetes

Table S5. Percent change (95% CI) or β (95% CI) in inflammatory biomarker concentrations per doubling of PFOS. Highlighted boxes signify p<0.05. β can be interpreted as standard deviation-change in the index for each doubling of PFOS. Models are adjusted for maternal age, pre-pregnancy BMI, education, race, parity, physical activity, smoking status at visit 3, gestational age at visit 3.

| **Biomarker** | **Main analyses (n = 1411)** | **Excluding participants (n=54) who regularly take anti-inflammatory medications** | **Excluding participants (n=38) with pre-eclampsia** | **Excluding participants (n=14) with prior T1D or T2D** | **Excluding participants (n=76) with prior IGT or GDM** |
| --- | --- | --- | --- | --- | --- |
| **Percent change (95% CI)** | | | | | |
| MIP-1ß | 1.05 (-1.33, 3.48) | 0.96 (-1.45, 3.44) | 0.88 (-1.49, 3.3) | 1.11 (-1.29, 3.56) | 1.14 (-1.29, 3.63) |
| MCP-1 | 2.68 (-0.39, 5.86) | 2.21 (-0.94, 5.45) | 3.07 (-0.05, 6.28) | 2.53 (-0.57, 5.72) | 2.54 (-0.59, 5.78) |
| TNF-α | 0.96 (-2.13, 4.15) | 0.84 (-2.3, 4.08) | 0.93 (-2.18, 4.13) | 1.03 (-2.09, 4.25) | 0.74 (-2.40, 3.97) |
| IFN-γ | -3.75 (-10.63, 3.67) | -3.27 (-10.32, 4.33) | -3.57 (-10.53, 3.93) | -3.44 (-10.41, 4.08) | -4.25 (-11.16, 3.19) |
| IL-2 | 4.62 (-4.09, 14.12) | 3.57 (-5.18, 13.12) | 4.8 (-4.04, 14.44) | 4.59 (-4.18, 14.16) | 3.95 (-4.85, 13.56) |
| IL-6 | -1.07 (-6.52, 4.7) | -1.74 (-7.21, 4.06) | -1.19 (-6.7, 4.64) | -0.84 (-6.36, 5) | -1.1 (-6.68, 4.81) |
| IL-8 | -0.53 (-4.2, 3.27) | -0.97 (-4.7, 2.9) | -0.4 (-4.1, 3.43) | -0.39 (-4.09, 3.45) | -0.33 (-4.04, 3.54) |
| IL-10 | 2.11 (-3.25, 7.75) | 1.8 (-3.63, 7.55) | 1.44 (-3.92, 7.1) | 1.81 (-3.57, 7.5) | 2.36 (-3.05, 8.09) |
| IL-12 | -5.41 (-13.51, 3.45) | -6.76 (-14.93, 2.19) | -4.69 (-12.9, 4.29) | -5.35 (-13.52, 3.58) | -5.71 (-13.86, 3.2) |
| CRP | 0.93 (-5.92, 8.27) | 1.08 (-5.9, 8.58) | 0.7 (-6.16, 8.07) | 1.36 (-5.54, 8.76) | -0.21 (-7.01, 7.1) |
| MMP-1 | -0.64 (-5.14, 4.09) | -0.75 (-5.33, 4.06) | -1.43 (-5.9, 3.25) | -0.26 (-4.82, 4.52) | -0.96 (-5.51, 3.81) |
| MMP-2 | -0.14 (-3.20, 3.03) | 0.12 (-3.00, 3.35) | -0.19 (-3.29, 3.02) | -0.05 (-3.12, 3.13) | -0.58 (-3.73, 2.66) |
| MMP-7 | -3.55 (-7.59, 0.67) | -3.36 (-7.48, 0.94) | -4.17 (-8.2, 0.04) | -3.47 (-7.55, 0.79) | -4.43 (-8.5, -0.18) |
| MMP-9 | **8.27 (2.48, 14.39)** | **9.44 (3.45, 15.77)** | **8.67 (2.78, 14.89)** | **8.46 (2.6, 14.64)** | **7.88 (1.99, 14.11)** |
| MMP-10 | -2.75 (-5.71, 0.31) | -2.91 (-5.94, 0.22) | -2.82 (-5.79, 0.25) | -2.75 (-5.74, 0.33) | -2.49 (-5.51, 0.61) |
| ICAM | -3.02 (-6.25, 0.33) | -3.38 (-6.68, 0.03) | -3.37 (-6.61, -0.01) | -3 (-6.26, 0.37) | -2.54 (-5.79, 0.82) |
| VCAM | **-2.46 (-4.65, -0.23)** | **-2.48 (-4.70, -0.21)** | **-2.68 (-4.88, -0.43)** | **-2.4 (-4.6, -0.15)** | **-2.4 (-4.62, -0.12)** |
| VEGF | 6.47 (-0.45, 13.86) | 6.68 (-0.42, 14.27) | 6.09 (-0.79, 13.45) | 6.17 (-0.79, 13.63) | **7.63 (0.51, 15.26)** |
| GMCSF | 0.01 (-6.95, 7.49) | -0.64 (-7.6, 6.84) | 0.3 (-6.75, 7.89) | 0.44 (-6.59, 8.01) | 0.13 (-6.94, 7.74) |
| **β (95% CI)^1^** | | | | | |
| Pro-inflammatory index | 0.23 (-0.042, 0.50) | 0.20 (-0.079, 0.48) | 0.25 (-0.03, 0.52) | 0.23 (-0.04, 0.51) | 0.20 (-0.08, 0.48) |

β – parameter estimate; 95% CI – 95% confidence interval; T1D – Type 1 diabetes; T2D – Type 2 diabetes

Table S6. Percent change (95% CI) or β (95% CI) in inflammatory biomarker concentrations per doubling of PFHxS. Highlighted boxes signify p<0.05. β can be interpreted as standard deviation-change in the index for each doubling of PFHxS. Models are adjusted for maternal age, pre-pregnancy BMI, education, race, parity, physical activity, smoking status at visit 3, gestational age at visit 3.

| **Biomarker** | **Main analyses (n = 1411)** | **Excluding participants (n=54) who regularly take anti-inflammatory medications** | **Excluding participants (n=38) with pre-eclampsia** | **Excluding participants (n=14) with prior T1D or T2D** | **Excluding participants (n=76) with prior IGT or GDM** |
| --- | --- | --- | --- | --- | --- |
| **Percent change (95% CI)** | | | | | |
| MIP-1ß | 1.48 (-0.31, 3.31) | 1.41 (-0.42, 3.28) | 1.39 (-0.4, 3.21) | 1.47 (-0.34, 3.3) | 1.5 (-0.33, 3.37) |
| MCP1 | **2.98 (0.65, 5.36)** | **2.60 (0.22, 5.03)** | **3.15 (0.81, 5.56)** | **2.77 (0.43, 5.16)** | **2.77 (0.4, 5.19)** |
| TNF-α | -0.44 (-2.73, 1.92) | -0.58 (-2.9, 1.8) | -0.33 (-2.64, 2.04) | -0.36 (-2.68, 2.01) | -0.47 (-2.8, 1.92) |
| IFN-γ | -3.93 (-9.14, 1.58) | -3.24 (-8.57, 2.41) | -3.76 (-9.03, 1.82) | -3.87 (-9.14, 1.69) | -3.28 (-8.56, 2.31) |
| IL-2 | 5.81 (-0.87, 12.94) | 5.69 (-1.08, 12.91) | **6.86 (0.01, 14.17)** | 5.72 (-1.00, 12.9) | 5.35 (-1.41, 12.58) |
| IL-6 | 1.78 (-2.46, 6.2) | 1.9 (-2.38, 6.38) | 1.64 (-2.65, 6.11) | 1.88 (-2.4, 6.35) | 1.76 (-2.58, 6.29) |
| IL-8 | -1.54 (-4.27, 1.28) | -1.67 (-4.45, 1.19) | -1.4 (-4.17, 1.44) | -1.55 (-4.31, 1.28) | -1.77 (-4.53, 1.07) |
| IL-10 | 0.4 (-3.57, 4.55) | 0.61 (-3.45, 4.84) | 0.32 (-3.7, 4.51) | 0.26 (-3.75, 4.43) | 0.17 (-3.84, 4.34) |
| IL-12 | -3.97 (-10.22, 2.7) | -3.78 (-10.17, 3.07) | -4.31 (-10.57, 2.39) | -4.21 (-10.48, 2.51) | -3.96 (-10.26, 2.78) |
| CRP | 1.52 (-3.7, 7.01) | 1.64 (-3.67, 7.23) | 2.09 (-3.19, 7.65) | 1.99 (-3.27, 7.53) | 1.3 (-3.92, 6.81) |
| MMP-1 | -0.16 (-3.58, 3.39) | 0.020 (-3.46, 3.63) | -0.59 (-4.01, 2.94) | 0.030 (-3.43, 3.61) | 0.3 (-3.19, 3.9) |
| MMP-2 | **2.98 (0.59, 5.42)** | **3.13 (0.7, 5.61)** | **2.8 (0.39, 5.28)** | **2.88 (0.49, 5.33)** | **2.71 (0.27, 5.22)** |
| MMP-7 | -1.15 (-4.29, 2.09) | -1.34 (-4.52, 1.95) | -1.25 (-4.41, 2) | -1.19 (-4.35, 2.08) | -1.53 (-4.7, 1.75) |
| MMP-9 | 2.58 (-1.58, 6.91) | 2.74 (-1.52, 7.18) | 2.83 (-1.4, 7.25) | 2.61 (-1.58, 6.99) | 2.12 (-2.1, 6.53) |
| MMP-10 | -0.95 (-3.23, 1.39) | -1.16 (-3.49, 1.23) | -1.06 (-3.35, 1.29) | -0.93 (-3.23, 1.42) | -0.88 (-3.19, 1.49) |
| ICAM | 2.29 (-0.28, 4.93) | 2.38 (-0.26, 5.09) | 2.24 (-0.36, 4.91) | 2.33 (-0.27, 4.99) | 2.63 (0.05, 5.27) |
| VCAM | 1.7 (-0.02, 3.44) | **1.85 (0.1, 3.62)** | 1.6 (-0.13, 3.37) | **1.76 (0.02, 3.51)** | **1.85 (0.1, 3.63)** |
| VEGF | 3.82 (-1.29, 9.2) | 3.76 (-1.47, 9.26) | 3.92 (-1.19, 9.31) | 3.71 (-1.45, 9.14) | 3.8 (-1.4, 9.29) |
| GMCSF | -0.77 (-6.00, 4.75) | -0.28 (-5.56, 5.3) | -0.28 (-5.6, 5.34) | -0.75 (-6.01, 4.8) | -0.42 (-5.73, 5.2) |
| **β (95% CI)^1^** | | | | |  |
| Pro-inflammatory index | **0.21 (0.01, 0.41)** | 0.19 (-0.017, 0.4) | **0.22 (0.01, 0.42)** | 0.20 (-0.0023, 0.41) | 0.19 (-0.021, 0.393) |

β – parameter estimate; 95% CI – 95% confidence interval; T1D – Type 1 diabetes; T2D – Type 2 diabetes

Table S7. Percent change (95% CI) in inflammatory biomarker concentrations per doubling of PFAS stratified by male (n = 742) and female (n = 668) fetal sex. Significant strata-specific associations indicated in yellow. Significant product term (p<0.1) indicated with asterisk (*). β can be interpreted as standard deviation-change in the index for each doubling of PFAS. Models are adjusted for maternal age, pre-pregnancy BMI, education, race, parity, physical activity, smoking status at visit 3, gestational age at visit 3.

|  | **PFOA** | | | **PFOS** | |  | **PFHxS** | |  |
| --- | --- | --- | --- | --- | --- | --- | --- | --- | --- |
| **Biomarker** | **Male (n=742)** | **Female (n=668)** | **Product term p-value** | **Male (n=742)** | **Female (n=668)** | **Product term p-value** | **Male (n=742)** | **Female (n=668)** | **Product term p-value** |
| MIP-1ß | 2.76 (-0.95, 6.61) | **6.00 (2.21, 9.92)** | 0.86 | 1.45 (-1.94, 4.95) | 0.58 (-2.73, 4) | 0.26 | 1.02 (-1.56, 3.66) | 2.31 (-0.19, 4.88) | 0.88 |
| MCP-1 | 4.27 (-0.37, 9.11) | 4.07 (-0.92, 9.32) | 0.85 | 0.63 (-3.49, 4.94) | **4.75 (0.15, 9.57)** | 0.20 | 2.14 (-1.05, 5.45) | **3.75 (0.35, 7.26)** | 0.69 |
| TNF-α | 2.00 (-2.78, 7.01) | -0.3 (-4.97, 4.59) | 0.65 | 1.42 (-2.97, 6.01) | 0.28 (-4.01, 4.78) | 0.64 | 0.01 (-3.31, 3.43) | -1.19 (-4.35, 2.08) | 0.59 |
| IFN-γ | 2.00 (-8.56, 13.78) | -1.16 (-12.5, 11.64) | 0.75 | -3.59 (-12.83, 6.64) | -4.51 (-14.55, 6.7) | 0.91 | -0.41 (-7.76, 7.53) | **-8.58 (-15.8, -0.74)** | 0.22 |
| IL-2 | 1.47 (-11.25, 16.01) | 8.58 (-5.19, 24.35) | 0.55 | 4 (-8.08, 17.67) | 6.02 (-6.32, 19.97) | 0.87 | **11.31 (1.37, 22.23)** | 0.43 (-8.39, 10.1) | 0.18 |
| IL-6 | -3.47 (-11.48, 5.27) | 6.33 (-2.71, 16.21) | 0.08 * | -1.49 (-9.07, 6.72) | 0.03 (-7.77, 8.51) | 0.74 | 1.22 (-4.76, 7.58) | 3.1 (-2.93, 9.51) | 0.58 |
| IL-8 | -1.00 (-6.33, 4.64) | 1.01 (-4.98, 7.39) | 0.60 | -2.04 (-6.92, 3.1) | 1.02 (-4.47, 6.84) | 0.51 | -1.56 (-5.32, 2.34) | -2.12 (-6.09, 2.02) | 0.81 |
| IL-10 | 3.24 (-4.82, 11.98) | -1.8 (-9.89, 7.01) | 0.09 * | 4.97 (-2.61, 13.14) | -0.17 (-7.69, 7.98) | 0.97 | **6.91 (1.00, 13.16)** | **-5.68 (-11.00, -0.04)** | 0.00078 * |
| IL-12 | -3.04 (-15.72, 11.56) | 3.03 (-10.07, 18.05) | 0.93 | -6.56 (-17.9, 6.35) | -3.49 (-14.78, 9.29) | 0.13 | -4.87 (-13.79, 4.98) | -2.5 (-11.09, 6.93) | 0.81 |
| CRP | -7.08 (-16.35, 3.22) | -1.21 (-11.74, 10.57) | 0.89 | -4.19 (-13.04, 5.56) | 5.23 (-5.05, 16.63) | 0.27 | 1.01 (-6.17, 8.73) | 1.12 (-6.32, 9.14) | 0.59 |
| MMP-1 | 3.4 (-3.49, 10.79) | -4.98 (-11.87, 2.45) | 0.05 * | 5.95 (-0.57, 12.9) | **-7.39 (-13.52, -0.84)** | 0.0034 * | 1.38 (-3.41, 6.41) | -1.12 (-6.04, 4.07) | 0.38 |
| MMP-2 | 1.52 (-2.44, 5.64) | **9.22 (3.13, 15.66)** | 0.11 | 0.12 (-3.49, 3.86) | -0.86 (-5.94, 4.5) | 0.67 | 1.51 (-1.29, 4.39) | **4.31 (0.32, 8.46)** | 0.47 |
| MMP-7 | -2.37 (-8.32, 3.97) | -1 (-7.76, 6.25) | 0.62 | **-5.66 (-10.97, -0.03)** | -1.42 (-7.57, 5.14) | 0.21 | -2.42 (-6.64, 2) | 0.24 (-4.45, 5.16) | 0.27 |
| MMP-9 | 5.57 (-2.42, 14.2) | 4.12 (-5.16, 14.31) | 0.72 | 6.23 (-1.19, 14.22) | **10.45 (1.47, 20.23)** | 0.63 | -2.06 (-7.32, 3.5) | **7.47 (0.91, 14.47)** | 0.081 * |
| MMP-10 | -0.87 (-5.3, 3.76) | -2.34 (-7.17, 2.73) | 0.64 | 0.12 (-4.02, 4.43) | **-6.32 (-10.52, -1.94)** | 0.08 * | -0.09 (-3.26, 3.19) | -1.94 (-5.24, 1.47) | 0.64 |
| ICAM | -1.38 (-6.17, 3.65) | -1.83 (-7.19, 3.85) | 0.17 | -0.79 (-5.24, 3.86) | **-5.49 (-10.19, -0.54)** | 0.17 | 2.88 (-0.64, 6.53) | 1.76 (-2.05, 5.7) | 0.68 |
| VCAM | -0.03 (-3.37, 3.43) | 0.17 (-3.42, 3.91) | 0.47 | -0.73 (-3.8, 2.43) | **-4.47 (-7.58, -1.25)** | 0.04 * | 1.21 (-1.18, 3.65) | 2.27 (-0.23, 4.83) | 0.98 |
| VEGF | 3.25 (-6.68, 14.23) | -0.86 (-10.94, 10.36) | 0.96 | 7.03 (-2.48, 17.48) | 5.95 (-3.91, 16.81) | 0.95 | 5.8 (-1.45, 13.57) | 1.92 (-5.22, 9.6) | 0.67 |
| GMCSF | 4.27 (-6.67, 16.5) | -2.96 (-13.25, 8.53) | 0.33 | 4.07 (-6.05, 15.28) | -3.61 (-12.98, 6.78) | 0.27 | 2.67 (-5.03, 10.99) | -3.89 (-10.91, 3.69) | 0.33 |
| **β (95% CI)^1^** | | | | | | | | |  |
| Pro-inflammatory index | 0.41 (-0.074, 0.89) | 0.30 (-0.021, 0.61) | 0.84 | 0.27 (-0.18, 0.72) | 0.16 (-0.13, 0.45) | 0.77 | 0.29 (-0.047, 0.63) | 0.10 (-0.11, 0.32) | 0.45 |

β – parameter estimate; 95% CI – 95% confidence interval.

Table S8. Geometric means (95% CI) for biomarkers stratified by fetal sex^a^.

|  | **GM (95% CI)** | |
| --- | --- | --- |
| **Biomarker** | **Male (n = 742)** | **Female (n = 668)** |
| MIP-1β (pg/mL) | 57.12 (55.54, 58.76) | 58.34 (56.78, 59.96) |
| MCP-1 (pg/mL) | 39.06 (37.72, 40.46) | 37.13 (35.80, 38.50) |
| TNF-α (pg/mL) | 4.49 (4.32, 4.65) | 4.28 (4.13, 4.43) |
| IFN-γ (pg/mL) | 4.25 (3.91, 4.62) | 3.92 (3.58, 4.29) |
| IL-2 (pg/mL) | 1.25 (1.13, 1.38) | 1.09 (0.99, 1.20) |
| IL-6 (pg/mL) | 1.75 (1.64, 1.87) | 1.68 (1.57, 1.80) |
| IL-8 (pg/mL) | 2.12 (2.03, 2.21) | 2.02 (1.93, 2.11) |
| IL-10 (pg/mL) | 21.96 (20.64, 23.36) | 19.94 (18.72, 21.23) |
| IL-12 (pg/mL) | 2.42 (2.17, 2.69) | 2.18 (1.97, 2.41) |
| CRP (μg/mL) | 17.05 (15.72, 18.49) | 16.98 (15.60, 18.49) |
| MMP-1 (ng/mL) | 0.69 (0.65, 0.72) | 0.72 (0.68, 0.76) |
| MMP-2 (ng/mL) | 67.23 (65.19, 69.32) | 68.17 (65.34, 71.12) |
| MMP-7 (ng/mL) | 5.74 (5.47, 6.03) | 5.71 (5.42, 6.02) |
| MMP-9 (ng/mL) | 28.59 (26,92, 30.37) | 27.69 (25.86, 29.65) |
| MMP-10 (ng/mL) | 0.26 (0.25, 0.27) | 0.26 (0.25, 0.27) |
| ICAM (ng/mL) | 142.51 (137.18, 148.05) | 143.00 (137.22, 149.03) |
| VCAM (ng/mL) | 251.03 (244.58, 257.66) | 249.14 (242.46, 256.01) |
| VEGF (pg/mL) | 2.35 (2.18, 2.55) | 2.26 (2.09, 2.45) |
| GM-CSF (pg/mL) | 1.29 (1.19, 1.41) | 1.15 (1.06, 1.25) |

^a^ Total sample is 1410 due to exclusion of one participant with unknown fetal sex.

GM – geometric mean; 95% CI – 95% confidence interval

CRP - C-reactive protein

GM-CSF - Granulocyte-macrophage colony-stimulating factor

ICAM - Intracellular adhesion molecule

IFN-γ - Interferon gamma

IL - Interleukin

MCP-1 - Monocyte chemoattractant protein-1

MIP-1ß - Human macrophage inflammatory protein-1 beta

MMP - Matrix metalloproteinase

TNF-*α -*Tumor necrosis factor alpha

VCAM - Vascular cell adhesion molecule

VEGF - Vascular endothelial growth factor

Table S9. Exposure, outcome, and participant characteristics between participants with 1^st^ trimester data, total sample, analytical sample, and women with missing covariate data.

|  | **Participants with 1^st^ trimester data (n=1886)** | **Total sample (n=1533)** | **Analytical sample (n=1411)** | **Participants with missing covariate data (n=122)** |
| --- | --- | --- | --- | --- |
| **GM (95% CI) (μg/L)** |  |  |  |  |
| PFOA | 1.65 (1.61, 1.70) | 1.67 (1.62, 1.72) | 1.66 (1.61, 1.71) | 1.74 (1.56, 1.93) |
| PFOS | 4.56 (4.45, 4.69) | 4.57 (4.43, 4.70) | 4.57 (4.43, 4.71) | 4.52 (4.11, 4.97) |
| PFHxS | 1.02 (0.99, 1.06) | 1.02 (0.98, 1.06) | 1.01 (0.97, 1.06) | 1.11 (0.95, 1.29) |
| **GM (95% CI)** |  |  |  |  |
| MIP 1ß (pg/mL) | - | 57.79 (56.72, 58.89) | 57.71 (56.59, 58.85) | 58.80 (55.08, 62.76) |
| MCP-1 (pg/mL) | - | 38.02 (37.11, 38.95) | 38.14 (37.19, 39.11) | 36.66 (33.73, 39.86) |
| TNF-*α* (pg/mL) | - | 4.37 (4.26, 4.48) | 4.39 (4.28, 4.50) | 4.15 (3.81, 4.51) |
| IFN-y (pg/mL) | - | 4.18 (3.95, 4.44) | 4.09 (3.85, 4.35) | 5.40 (4.45, 6.56) |
| IL-2 (pg/mL) | - | 1.16 (1.09, 1.25) | 1.17 (1.09, 1.26) | 1.10 (0.87, 1.40) |
| IL-6 (pg/mL) | - | 1.74 (1.66, 1.82) | 1.72 (1.64, 1.80) | 2.03 (1.70, 2.42) |
| IL-8 (pg/mL) | - | 2.07 (2.00, 2.13) | 2.07 (2.01, 2.14) | 2.02 (1.82, 2.26) |
| IL-10 (pg/mL) | - | 21.07 (20.21, 21.97) | 20.98 (20.08, 21.93) | 22.12 (19.44, 25.17) |
| IL-12 (pg/mL) | - | 2.33 (2.17, 2.50) | 2.30 (2.14, 2.48) | 2.67 (2.14, 3.33) |
| CRP (μg/mL) | - | 17.28 (16.33, 18.28) | 17.01 (16.04, 18.04) | 20.71 (16.96, 25.30) |
| MMP-1 (ng/mL) | - | 0.71 (0.68, 0.73) | 0.70 (0.67, 0.73) | 0.77 (0.68, 0.86) |
| MMP-2 (ng/mL) | - | 67.05 (65.44, 68.70) | 67.68 (65.96, 69.45) | 60.18 (56.30, 64.32) |
| MMP-7 (ng/mL) | - | 5.73 (5.54, 5.93) | 5.73(5.53, 5.94) | 5.80 (5.11, 6.58) |
| MMP-9 (ng/mL) | - | 28.47 (27.26, 29.74) | 28.18 (26.93, 29.48) | 32.01 (27.42, 37.57) |
| MMP-10 (ng/mL) | - | 0.26 (0.26, 0.27) | 0.26 (0.26, 0.27) | 0.25 (0.23, 0.28) |
| ICAM (ng/mL) | - | 143.86 (140.07, 147.74) | 142.77 (138.83, 146.82) | 157.03 (143.99, 171.25) |
| VCAM (ng/mL) | - | 250.77 (246.28, 255.33) | 250.19 (245.53, 254.93) | 257.51 (241.01, 275.14) |
| VEGF (pg/mL) | - | 2.32 (2.20, 2.45) | 2.31 (2.19, 2.44) | 2.45 (2.05, 2.94) |
| GM-CSF (pg/mL) | - | 1.23 (1.16, 1.30) | 1.22 (1.15, 1.30) | 1.36 (1.12, 1.65) |
| **Characteristic (%)** |  |  |  |  |
| > 30 years of age | 67.8 | 61.9 | 62.8 | 51.6 |
| White | 81.9 | 84.3 | 84.8 | 80.0 |
| Normal pre-pregnancy BMI | 54.4 | 56.6 | 61.1 |  |
| Never smoked | 53.2 | 61.5 | 61.7 | 59.0 |
| Nulliparous | 42.8 | 44.2 | 44.2 | 45.1 |
| Physical activity (>60 mins/day) | 48.4 | 50.0 | 50.1 | 48.4 |
| Education (Undergraduate degree or more) | 60.4 | 62.9 | 63.9 | 51.6 |

GM – geometric mean; 95% CI – 95% confidence interval

PFOA - Perfluorooctanoic acid

PFOS - Perfluorooctane sulfonate (PFOS)

PFHxS - Perfluorohexane sulfonate

CRP - C-reactive protein

GM-CSF - Granulocyte-macrophage colony-stimulating factor

ICAM - Intracellular adhesion molecule

IFN-γ - Interferon gamma

IL - Interleukin

MCP-1 - Monocyte chemoattractant protein-1

MIP-1ß - Human macrophage inflammatory protein-1 beta

MMP - Matrix metalloproteinase

TNF-*α -*Tumor necrosis factor alpha

VCAM - Vascular cell adhesion molecule

VEGF - Vascular endothelial growth factor

**SUPPLEMENTARY FIGURES**

**
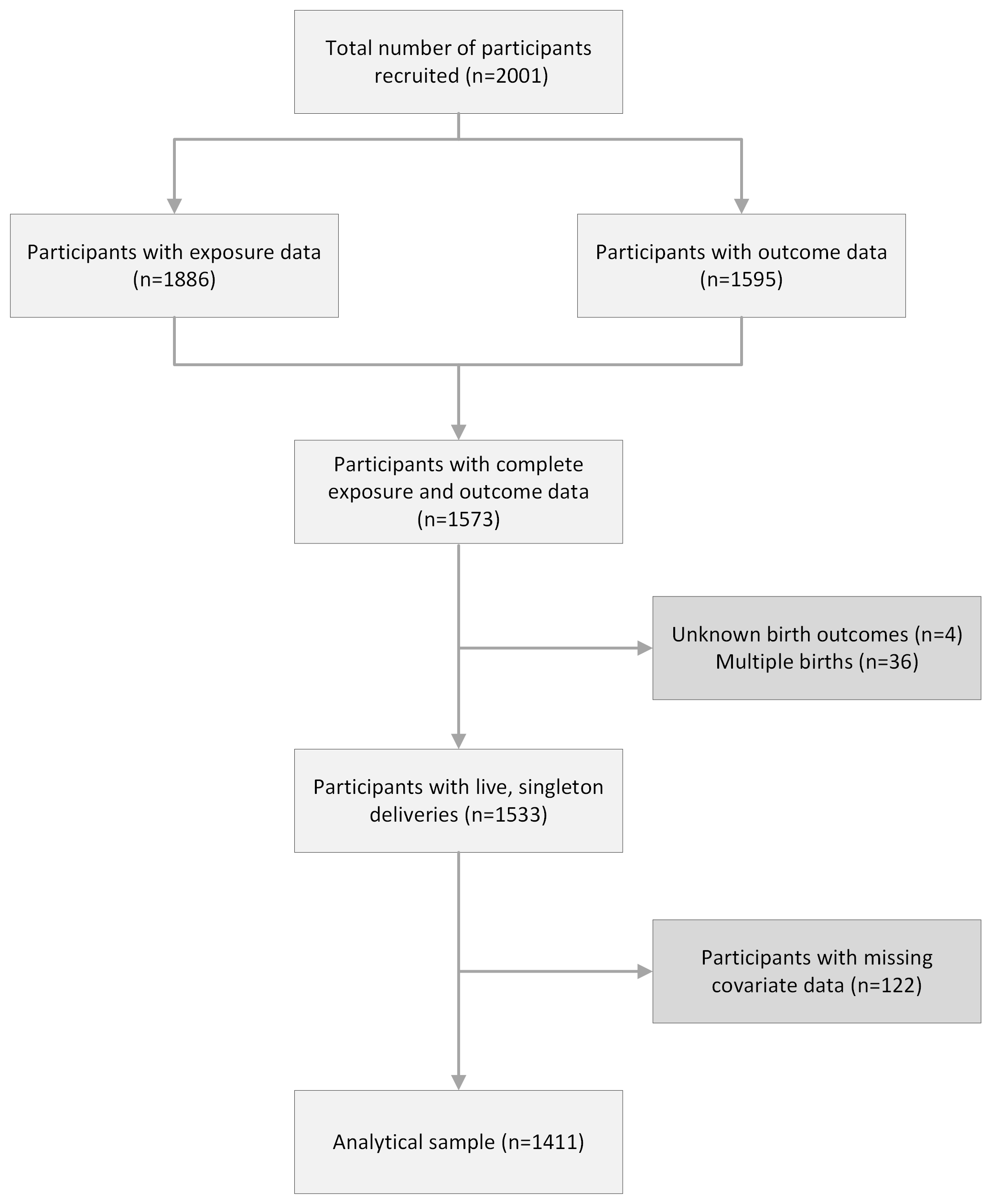
**

*Figure S1. Participant flow diagram displaying sample sizes for individual biomarker analyses.*

Figure S2. Directed acyclic graph used to identify important covariates in relationships of interest.


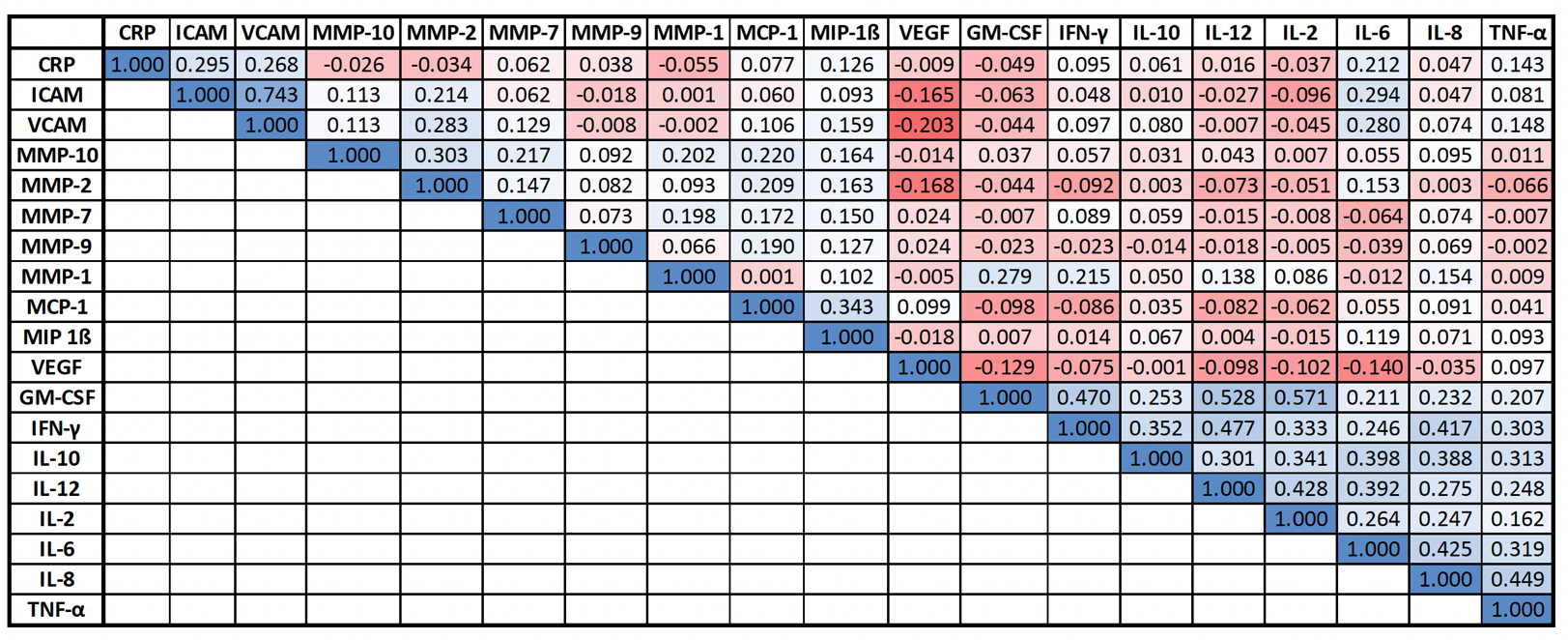
Figure S3. Spearman correlational coefficients of inflammatory biomarkers (n=19). Red gradient signifies a negative correlation and blue gradient signifies a positive correlation.
